# Report on the Meteorological Changes and More Important Diseases Prevalent in Chicago during the Summer of 1856

**Published:** 1857-01

**Authors:** N. S. Davis


### ARTICLE II.—*Report on the Meteorological Changes and More Important Diseases Prevalent in Chicago during the Summer of* 1856. By N. S. Davis, M.D. &c.

[Continued from the November Number.]

### FEVERS.

So far as my observation extended, very few attacks of fever of any variety occurred during the months of April, May, and

June, 1856. From the last week in the latter month, however, I began to meet more frequently with cases of simple remittent fever, and they continued to increase in number up to the end of July. From the 15th to the 31st of the latter month, I find recorded thirty cases of this variety of fever. Most of these were mild, uncomplicated, and required nothing peculiar in in their treatment. In five or six cases, occurring during the latter part of July, quinine in anti-periodic doses was not well borne, producing much cerebral disorder, and failing to fully cut short the febrile exacerbations. In all such, recourse was had to the ext. of cornus Florida with prompt success. I usually exhibited the extract in the form of pill, as follows, viz.:

℞—Ext. Cornus Florida, . ʒj.

Ext. Hyosciamus, . . ℈j.

Mix. Divide into twenty pills.

Give one every four hours until the exacerbations cease, and then only three times a-day until convalescence is fully established.

When the case was complicated with irritability of the mucous membrane of the intestines, from half a grain to one grain of opium was substituted for the hyosciamus in the pill. On the other hand, when the bowels were inactive, the secretions deficient, and the tongue covered with a thick coat, the ext. of cornus Florida was combined with a grain each of ext. of colocynth and blue mass, until a more healthy secretory action was established.

On the 2d day of August I was called to three cases of fever, presenting more or less resemblance to scarlatina. They were in children, varying from five to twelve years of age, and residing in the north-western part of the city. The symptoms were, a hot and dry skin, rapid pulse, much thirst, a whitish coat upon the tongue, urine scanty, bowels not disturbed, the glands of the neck near the angle of the jaws much swollen, and the fauces slightly red and tender. The greater part of the cutaneous surface was covered with a fine red rash. The rash, however, was more distinctly papular, and accompanied by less diffused general redness than in ordinary scarlatina: it was more irregular, also, in the time of its appearance. In two of the cases it was observed, on the first day, that the patients complained of being sick, while in the other it did not appear until the fourth day.

Under the influence of a mild alterative and diaphoretic treatment, with the external application of an infusion of aconite leaves and muriate of ammonia to the swollen glands, two of these patients rapidly recovered. The third continued to have some fever for two weeks, and the swollen glands continued large and indurated for twice that time.

During the first ten days of August I saw several other cases similar to the three just described. Some of them occurred in subjects who had previously had scarlet fever. This fact, together with the miliary appearance of the eruption and the irregularity as to the time of its development on the skin, led me to regard these cases as mild typhus accompanied by more than the usual amount of eruption, rather than true scarlatina.

At the same time that these eruptive cases were observed, chiefly in young subjects, a change began to be apparent in the character of nearly all the cases of fever that came under my observation. Those which had began with distinct paroxysms of remittent fever, if not arrested during the first three or four days, very generally put on the symptoms of the typhoid or typhus varieties.

The number of attacks of continued fever increased slowly through the whole month of August, while those of a true periodical or malarious character almost entirely ceased to recur.

During the whole month of September, continued fever was emphatically the *prevailing* disease in the city, the most malignant and fatal cases occurring between the 15th and 30th of that month. Since the 1st of October the disease has rapidly diminished, both in the frequency and severity of the attacks.

If my own observations were correct, the continued forms of fever did not prevail equally in all parts of the city at the same time. They were decidedly the most prevalent in the West Division during the last half of August, but they did not reach their greatest prevalence in the South Division until after the middle of September. I have no means of ascertaining the whole number of attacks during the season, and consequently could not give the ratio of mortality. The number of deaths from these forms of fever, as reported by the City Sexton, were as follows, viz.:—

| | |
|---|---|
| January, 1856, | 2 |
| February, | 5 |
| March, | 2 |
| April, | 3 |
| May, | 2 |
| June, | 1 |
| July, | 4 |
| August, | 12 |
| September, | 25 |
| October, | 23 |
| November, | 14 |

These figures cannot be relied on as absolutely correct, but they are sufficiently so for practical purposes. More deaths, however, are included in the month of October than belong properly to it, because a number of cases beginning in September did not terminate fatally until the first and second weeks of October, when new attacks had become less frequent. A large majority of those attacked were in the middle period of life, and residents of the city less than eighteen months. But no age or period of life was wholly exempt, and several of the most severe cases occurred in persons who had been constant residents for several years. In most cases, the first or forming stage was protracted through from four to six days, during which the patients complained of lassitude, a sense of weariness, dulness and lightness in the head, sometimes pains in the back and limbs, with slight alternations of heat and cold. During the month of August most of the cases presented even in the forming stage more or less disturbance of the alimentary canal, such as a sense of fulness, rumbling of gases, occasional slight pains, and from one to three or four thin fecal discharges every twenty-four hours. These cases, when fully developed, presented all the phenomena and tendencies of ordinary enteric typhoid fever.

So early, however, as the first week in August, and coincident with the cases of miliary fever already alluded to, a few cases of continued fever were observed without any enteric disturbance, but with all the marks of genuine typhus. The forming stage was protracted generally, and characterized by aching pains in the head, back, and limbs; either a normal or slightly inactive state of the bowels; loss of appetite; tongue covered with a dirty white coat, and mouth often tasting bitter with unnatural dryness; skin dry and harsh; the sleep at night disturbed and unrefreshing; and the mind dull and desponding. After these symptoms had continued from two to five or six days, decided rigors or chilliness would supervene, followed by decided increase of pain in the head and back, often accompanied by a sense of giddiness or confusion, especially when in the upright posture; a frequent, quick, but compressible pulse; hot and dry skin; dryness of the mouth, with thirst; a slight sense of soreness, with redness of the fauces, and an occasional cough, with a feeling of fulness or oppression in the chest; a moderate degree of redness or suffusion of the face, coupled with a watery appearance of the eyes and a dull or gloomy expression of countenance; and mental torpor or drowsiness, with entire inability to procure refreshing sleep. Though the bowels were in most of these cases normal or slightly constipated during the first few days, they were generally easily acted upon by medicine possessing cathartic properties, and sometimes to an inordinate degree. When not materially modified by treatment, this class of cases pursued a pretty uniform course. The pulse remained frequent, moderately full, but soft; the skin more dry; the countenance more dull, and suffused with a deeper or darker redness; the tongue covered, particularly along the middle, with a thick reddish-brown and dry coat; the respiration shorter than natural, the inflation of the lungs incomplete, and the cough, though not frequent or much noticed by the patient, yet generally harsh and accompanied by only slight expectoration. If resort was had to physical exploration, very few cases were found to reach the end of the first week after the patient took to his bed, without presenting increased dulness over the inferior and posterior portions of one or both lungs, with great feebleness or entire suppression of the respiratory murmur, and more or less submucous rhoncus in the same regions, while over the upper and anterior part of the chest the dry bronchial rales were prominently developed, and in many cases persistent throughout the whole course of the disease. In the meantime the mind grew daily more dull and disposed to incoherence or low delirium, especially during the night. If the disease was still unmodified by treatment, all the symptoms slowly but steadily increased; and, at the end of the second, or during the third week, the mouth and tongue were seen to be entirely dry, the latter difficult of protrusion and more or less tremulous; the lips dry and of a purplish red color, the upper one retracted, leaving the upper teeth exposed and covered with brown sordes; the face in some was still flushed with a dark livid redness, in others it presented a dingy or ashy paleness; the mind almost constantly somnolent, or in a state of low, wandering delirium, though capable of being roused so as to answer questions correctly; the voice was gruff and altered, the speech slow and hesitating, the patient sometimes becoming lost and stopping in the middle of a sentence; the deglutition awkward and sometimes gurgling; the hearing obtuse; the action of the voluntary muscles slow and unsteady, with more or less subsultus tendinum; the urine in most cases scanty and high colored, though in some it was filled with a whitish deposit, yielding a strongly offensive or ammoniacal smell, and in a few instances it was retained in the bladder until removed by the catheter. In one unusually protracted and severe case I was obliged to use the catheter daily for more than a week. The bowels in most cases remained quiet unless disturbed by medicine; but mild cathartics almost always acted promptly, and were generally followed by a looseness amounting to three or four thin and brownish stools daily, for two or three days, or until checked by anodynes, and the abdomen was moderately full, but seldom very tympanitic. The state of the abdomen, however, varied very much in different cases. In almost every case that came under my care, in which active cathartics had been given in the early stage, the abdomen became more tympanitic as the disease advanced; the bowels decidedly loose, and the tongue unnaturally red and dry. In most cases the stomach was quiet and retentive throughout the whole course of the disease; but in a few it was sufficiently irritable to cause the prompt rejection of food and drinks unless taken in very small quantities. In a small number of cases that came under my observation, a very troublesome disposition to vomit came on in the advanced stage of the disease; the matter ejected being chiefly a thin serous fluid, more or less green or bluish in color. This was not only a troublesome symptom of itself, but it was almost always accompanied by a dangerous degree of prostration.

In this advanced stage, the indications of engorgement and obstruction of the lungs became plainer and of more serious import. The lateral and posterior parts of the chest were found more dull on percussion, and in the same regions the respiratory murmur was superseded by a sub-mucous or sub-crepitant rale, and at some points almost entire silence existed. The expansion of the chest was feeble and imperfect, often very unequal on the two sides. The cough continued deep and severe, but not of frequent recurrence; and in most cases the expectoration remained scanty and composed of a tenacious, whitish, or dirty-white mucous.

In these cases the patients rarely complained of either pain or soreness in the chest; and, aside from the occasional annoyance which the cough gave them, they seemed unconscious of any serious difficulty with the respiratory organs. In a smaller number of cases, however, the symptoms and signs were such as to indicate the existence of more decided pneumonic inflammation. The patient, if not too somnolent or delirious, complained of some pain, a sense of oppression and soreness in the mammary, and some times as high up as the infra-clavicular, regions. The cough was more frequent, and accompanied by more or less bloody expectoration, generally of a dark red color. Dulness, on percussion, was found higher up, and more anteriorly than in the cases of simple pulmonary engorgement. Auscultation easily detected the existence of a sub-crepitant rale in some places; a mingling of moist and dry rales in others, with increased vibration of voice or diffused bronchophony.

In two protracted cases that came under my care in the fourth week of their progress, the patients presented, in addition to the preceding symptoms of extensive pulmonary engorgement or partial hepatization, a very deep yellow or jaundiced hue of the conjunctiva and whole cutaneous surface, with a free expectoration of a bright orange-yellow color.

In a large proportion of the fatal cases of typhus or typhoid fever, which have occurred during the past summer and autumn, the immediate cause of death has been the extent of the pulmonary lesions. In such, the fatal result was generally preceded one or two days by a dingy or leaden paleness of the countenance, a purple hue of the prolabia, a dull or lethargic condition of mind, a frequent and feeble pulse, more or less subsultus, some difficulty of deglutition, or rather each attempt to swallow produced a paroxysm of coughing, with a sense of partial strangulation and great weariness; the breathing short and noisy, from the rattling of mucous in the larger bronchial tubes; and the extremities cool, with much feebleness of capillary circulation in the cutaneous surface. In three cases that came under my observation, life was cut short by a rapid hemorrhagic infiltration of the lungs, causing the patient to commence suddenly the expectoration of large quantities of a thin, dark-colored, bloody mucus, which accumulated so much faster than they were able to expectorate it, that complete suffocation was induced in a few hours. One of these cases I saw in consultation with a neighboring physician only a few hours before the patient died. The attending physician assured me, that, though the patient had been laboring under the fever three or four weeks, he had not noticed evidences of any serious determination to the lungs until the last two days. The other two cases occurred in the Mercy Hospital, and were admitted too near the fatal result to obtain a reliable history of their preceding symptoms.

In the cases of continued fever, which occurred between the 20th of September and the 15th of October, there was noticed an unusual tendency to hemorrhage from the bowels, accompanied by sudden and dangerous prostration. During the time specified, I saw not less than eight cases which presented this symptom in a greater or less degree. Some of these were in the Mercy Hospital, and others were merely seen in consultation with other physicians. The only case of this kind that proved fatal in my private practice was the following, which we give in detail:—

Mr. P. L. aged about fifty years, excessively fleshy, or of a strongly lymphatic temperament, and addicted to the free use of alcoholic beverages, called at my office on the 10th of October. On careful inquiry I learned that he had been several days complaining of pains in his head, back, and limbs, with the usual accompanying symptoms of fever. He had, on the 8th, taken some cathartic medicine, which operated quickly and excessively, procuring numerous thin serous evacuations. His face was deeply flushed; his lips dry and red; his tongue coated with a white fur and rather dry; his pulse one hundred and ten per minute, full, but soft; his skin dry and hot; and his breathing asthmatic. He complained of much thirst, a sense of fulness and rumbling of gases in his bowels, with too frequent and serous discharges; pains in his limbs; and a sense of lightness, giddiness, or confusion in his head. His sleep during the preceding night had been very unquiet, and his mind somewhat wandering. The chest was clear on percussion, but auscultation revealed a loud and dry ronchus over both sides, composed of a mingling of the sonorous and sibilant or hissing sounds, and indicating a dry and congested condition of the pulmonary mucous membrane.

I directed the patient to return home, and keep entirely quiet; and, as he had taken a mercurial cathartic which had operated excessively, leaving the bowels still irritable, I directed the following, viz.:—

℞—Ol. Terebinth, . . ʒij.

Tinct. Opii, . . ʒij.

Pulv. G. Arabac, }

White Sugar, } aa ʒiij.

Rubbed thoroughly together and added.

Mint Water, . . . ℥ij.

Of which he was to take one tea-spoonful every four hours.

To lessen the febrile excitement and promote a more free secretion from the pulmonary mucous membrane, I also directed the following, viz.:—

℞—Hive Syrup, . . . ℥j.

Tinct. Opii et Camph. . ʒjss.

Tinct. Verat. Viride, . . ʒj.

Mix,

And give one tea-spoonful between each of the doses of emulsion.

On the 11th I visited the patient at his residence, nearly three miles distant. His face was less flushed, skin less hot and dry, his pulse about ninety per minute and soft, and he expressed himself as feeling much better. Still his breathing was oppressed, his lips a little purple, a strip on the middle of his tongue dry, and his abdomen was full and hard, though no alvine discharges had occurred since the preceding day. His hearing was obtuse, and his mind dull, with much wandering or delirium during the preceding night. Not wishing any further sedative influence from the veratrum, and fearing an increase of the pulmonary congestion from too long a suppression of the intestinal discharges, I directed both the prescriptions of the preceding day to be omitted, and in their place left three powders, each containing, calomel three grains, and bi-carb. of soda five grains, to be given at intervals of once in four hours, and if they failed to operate on the bowels, they were to be followed by a table-spoonful of castor oil and twenty drops of oil of turpentine. On the other hand, if the bowels should be moved too freely, the preceding emulsion of turpentine and laudanum was to be resumed, and repeated at intervals of every two hours until they were again quieted.

The next morning a messenger called, requesting me to visit the patient without delay. On my arrival, I found him presenting much the same symptoms and appearance as on the preceding day, except that his skin was covered with warm perspiration, his tongue more moist, and his pulse one hundred per minute and softer.

After he had taken two of the powders of calomel and soda, directed for him yesterday, and before the hour for taking the third, he had two copious evacuations from the bowels, consisting almost wholly of blood. I saw the last one, which consisted of nearly a quart of dark, grumous, and partially-coagulated blood, without any intermixture of fæcal matter. On account of these discharges, the third powder was not given, but in its place the patient had taken a tea-spoonful of the emulsion of oil of turpentine and laudanum every two hours.

Though the hemorrhage was now apparently checked, yet the relaxed and moist skin, the moist tongue, the purplish hue of the prolabia, the dulness of intellect and special senses, with a continuance of the tumid and tympanitic state of the abdomen, all indicated a condition favorable to a recurrence of the hemorrhage. Hence I directed a continuance of the emulsion every four hours, and a powder consisting of, tanate of quinine five grains, and pulv. opii one grain, half-way between.

On the 13th, I found the patient with the skin and tongue dryer; the mental wandering and dulness of hearing somewhat increased; the breathing constricted and heavy; and the abdomen more full and tense than on the preceding day. There had been no evacuations of any kind from the bowels, and only a scanty secretion of urine. I now directed the exhibition of half an ounce of castor oil, with half a drachm of oil of turpentine and eight drops of laudanum. The same to be repeated in four hours, if the first did not operate. On the other hand, if it should operate too freely, the emulsion and the powders of quinine and opium were to be resumed, and aided, if necessary, by an anodyne and astringent enema.

On the 14th, I found the patient improved in every respect, except the dulness of intellect and disposition to low, wandering delirium. The oil had procured three large evacuations, consisting of dark-colored, semi-fluid, and offensive fæcal matter, without any intermixture of blood. I directed the emulsion of turpentine and laudanum to be continued every four hours, alternated with a powder composed of, sulph. quinine two grains, pulv. gum camphor two grains, pulv. opium one grain, mixed; and beef-tea for nourishment.

The patient continued comparatively comfortable, without any intestinal evacuations, and but little symptoms of fever, until about noon of the 15th, when he was again suddenly affected with a return of the hemorrhage. I saw him about four hours later, at which time he had had three or four copious discharges, consisting entirely of dark blood, and was much prostrated. Stimulants, coupled with anodynes and strong astringents, were promptly administered, both by the mouth and the rectum, but without arresting the hemorrhage, and the patient died during the succeeding night.

Another very unpleasant complication, which I saw in six of the cases of continued fever that came under my care during the past autumn, consisted of a peculiar inflammation and swelling of the parotid gland and adjacent cellular tissue. Four out of the six occurred in patients in the Mercy Hospital. This affection does not generally make its appearance until the end of the second, or during the third week of the fever. In one of the patients just alluded to, however, the parts under the ear and behind the angle of the jaw began rapidly to swell on the sixth day after the development of the fever. In this case, as in several others that came under my observation during the summer, the first onset or development of the fever was accompanied by a severe sense of soreness in the fauces, rendering the deglutition both difficult and painful. On examination, the whole fauces were seen to be very red, and the tonsils moderately swollen. The patient complained of pains in the head and limbs, with a hot and dry skin; a white coat on the tongue; and a pulse moderately full, and one hundred and ten per minute. A strong solution of the nitrate of silver was applied to the fauces with a sponge, and afterwards a gargle of chloride of zinc, one grain to the ounce of water. Internally, he took a powder every four hours, consisting of, pulv. Doveri six grains, sub-murias hydrarg. two grains, and pulv. nitrate of potassa five grains; with a tea-spoonful of spirits of nitre dulcis between. At the end of twenty-four hours after the commencement of the treatment, his bowels were opened by castor oil, after which he was directed to take a tea-spoonful of an expectorant mixture, consisting of hive syrup, tinct. blood-root, and camph. tinct. of opium.

But, feeling his throat some better, very unexpectedly and injudiciously left the hospital. At the end of three days he was brought back with an increased soreness of his fauces; a dark red flush on the face; a hot and dry skin; pulse one hundred and twenty per minute, and small; a frequent and harsh cough; a loud and dry bronchial ronchus over the upper part of both sides of the chest; tongue covered with a brown coat, dry in the middle; bowels moderately loose; and a swelling of the parotid gland, with all the adjacent tissues, on the right side of the neck, extending from the ear behind the angle of the jaw down nearly to the middle line under the chin. The swelling was most prominent over the parotid gland, exhibiting an undue degree of redness, and very hard to the touch. Within forty-eight hours from this second admission, the same kind of swelling of the parotid gland of the left side also occurred and increased rapidly, rendering the patient incapable of separating his jaws or receiving anything into his mouth without great difficulty.

On the fourth day it was evident, from the more purple color, the softer and more doughy feel of the tumor behind the angle of the jaw on the right side, that suppuration with gangrene of the cellular tissue had commenced. The patient also began to exhibit signs of greater prostration. His skin was cool; his pulse one hundred and thirty per minute, and small; his hearing obtuse; his mind wandering; his voluntary muscles unsteady and trembling; with four or five thin and brown fæcal discharges within the twenty-four hours. From this time the patient continued to sink rapidly, and died on the seventh or eighth day after the second admission.

The other five cases in which this peculiar swelling of the parotids occurred, recovered; but not without a protracted convalescence.

Careful examination was made in nearly all the cases of continued fever that came under my care, concerning the appearance of cutaneous eruptions. In about one-third of the cases, a fine red rash was observed on the chest and abdomen during the second week of the fever. In a few instances the rash was copious, and extended to the neck and arms: in others, it was so slight as to be easily overlooked. A few *rose*-colored spots were seen on the abdomen of three enteric cases that came under my care early in August.

The most striking peculiarities of the continued fevers of the past summer and autumn, were the extreme frequency of pulmonary complications, and the predominance of the symptoms of typhus as distinguished from the enteric typhoid fever. The first of these peculiarities will be strikingly shown by the following figures, viz.:—During the four months of August, September, October, and November, there came under my care, in private practice, sixty cases of typhoid and typhus fevers, thirty-eight of which presented well-marked indications of either congestion or inflammation in one or both lungs. During the three months ending with the 30th of November, there were admitted into the medical wards of the Mercy Hospital forty-six cases of typhoid and typhus fevers, viz., in September twenty-five, October twelve, and November nine. Of these cases twenty-nine presented all the signs, physical and rational, of engorgement or inflammation of the lower and posterior parts of both lungs, and five others of only one lung, making a total of thirty-four out of forty-six cases. This feature of these fevers was doubtless owing to the unusually low temperature of July, August, and September, as described in the first part of this report.

Notwithstanding the peculiarities just alluded to, the fevers of the past summer do not appear to have presented a high ratio of mortality. Thus, of the whole number of cases occurring in my private practice, not including those merely visited in consultation with other physicians, four proved fatal, being in the ratio of one in fifteen. Of the forty-six admitted into the Mercy Hospital during the three months previously named, seven died, viz.:—Two during the first day after admission, one the second, one the third, one the fourth, one the fifteenth, and one the nineteenth. It is thus seen that five out of these seven fatal cases were admitted with the disease already so far advanced as to afford little or no opportunity for the application of remedial measures. One of the fatal cases which occurred in private practice has already been given in detail. Another was a most estimable lady, aged about thirty years, who had resided several years in the city, and who was attacked severely with symptoms of typhus about the middle of September. From the first, her pulse was between one hundred and ten and one hundred and twenty per minute, and small; and she constantly complained of a peculiar sense of distress and oppression in the region covered by the lower half of the sternum. At first, her fever was paroxysmal, presenting so distinct a remission each morning, that, after having given alteratives and diaphoretics for one or two days, I gave her efficient antiperiodic doses of quinine each morning, for two successive mornings, but without any beneficial effect. She was then kept on a mild sedative and diaphoretic course of treatment, under which she gradually improved, until, at the end of the second week, her pulse was not more than eighty-five per minute, skin soft and normal in temperature, tongue clean and moist, and a slight return of appetite. Still she complained of an unusual sense of weakness or bad feeling behind the lower part of the sternum. At this stage of her progress, she was attacked with a severe gangrenous inflammation within the mouth. It was developed so suddenly, that, during a single night, the part of the cheek opposite the junction of the two maxillary bones, and that covering the ramus of the lower jaw, became so tumefied as to present in the morning a swollen and peculiar shining aspect externally. At the same time the inside of the cheek was much tumefied, of an ash-grey color, and the patient unable to open the jaws sufficiently to allow an examination of the fauces. The disease was limited exclusively to the back part of one cheek and the junction of the jaws: all the rest of the mouth and gums appeared perfectly healthy. Within twenty-four hours after the first appearance of disease in the cheek, the inner surface became entirely gangrenous and of a brown color. By a judicious use of tonics the gangrene soon ceased to spread —but the dead separated from the living parts very slowly, keeping the breath constantly impregnated with a putrid odor, which the most faithful application of disinfectants could only partially remedy. Still the patient continued to progress favorably for six days, when there occurred during the night copious showers, with unusually loud peals of thunder. The effect of this terrific thunder was very perceptible on several patients. On the lady in question, it produced much nervous excitement, coupled with partial delirium; a pulse of one hundred and thirty per minute, small and feeble; and two copious alvine evacuations, one of which was only partially under the patient's control. Although the tendency to diarrhœa was speedily checked by the emulsion of turpentine and laudanum, and the nervous excitement overcome by camphor and hyosciamus, yet her pulse continued feeble, the face pale, and the extremities cool. At the end of forty-eight hours the external swelling on the side of the face extended farther down upon the side of the neck; the skin over the part originally attacked became of a purplish color; the deglutition more difficult, and more constant subsultus tendinum. It was evident that the gangrenous affection had been renewed, and extended to the tonsil and side of the throat. Under the influence of strong stimulants and tonics she continued to live four days longer. She died on the twentieth day from the commencement of the disease.

The remaining two fatal cases which occurred in private practice, belonged to the poorer classes, and had been almost entirely neglected during the first two weeks of the fever. They both died from extensive engorgement of the lungs, accompanied by excessive intestinal discharges; the latter having been induced by the injudicious use of active physic.

To enter upon the details of the treatment adopted in the different cases and stages of continued fever during the past season, would extend the report much beyond its appropriate length. When called soon after the patient had been compelled to take his bed, and finding the pulse over one hundred per minute, and moderately full; the skin dry and hot; the face suffused with redness; and the tongue covered with a white or slightly-brownish coat, but without any special tendency to diarrhœa or pulmonary engorgement, I generally prescribed a powder consisting of, sub-murias hydrarg. two grains, pulv. Doveri five grains, every four hours, with a tea-spoonful of the following mixture between, viz.:—

| | | |
|---|---|---|
| ℞—Spts. Nit. Dulc. | . . | ℥j. |
| Tinct. Opii et Camph. | . | ℥j. |
| Tinct. Verat. Viride, | . | ʒj. |
| Mixed. | | |

The dryness and heat of the skin was much diminished also by frequent bathing or sponging over the surface with cold water, and the application of cold cloths to the head. These remedies, continued from twenty-four to thirty-six hours, generally reduced the pulse below ninety per minute, and caused a decided diminution of all the febrile symptoms. When this occurred without any action on the bowels, the remedies were suspended long enough to procure two or three evacuations by means of castor oil, or the Rochelle salts given with the effervescing draught. The veratrum mixture was then resumed, but in rather smaller doses, and only one powder of calomel and Dover's powder given in the evening. By these means alone convalescence was established in several apparently severe cases before the end of the first week after commencing the treatment. In many other cases, however, the alterative powders, alternated with the veratrum mixture, not only reduced the pulse and abated all the other febrile symptoms, but before the end of the twenty-four or thirty-six hours they induced copious thin evacuations from the bowels, sometimes coupled with vomiting. In such cases these remedies were immediately suspended, and the excessive irritation of the stomach and bowels allayed by anodynes, coupled with such medicines as are calculated to restore a more healthy action in the mucous membranes. One of the best combinations for this purpose, especially when the stomach is much nauseated, is the following, viz.:—

| | |
|---|---|
| ℞—Sulphas Magnesia, . . | ʒij. |
| Tinct. Opii, . . . | ʒij. |
| Aromat. Sulph. Acid, . | ʒij. |
| Water, . . . | ℥ij. |
| Mix, | |

And give a tea-spoonful every two or three hours, diluted with water.

In one severe case of fever, the exhibition of the small doses of calomel and Dover's powder, alternated with the veratrum, was followed, at the end of twenty-four hours, by excessive nausea and frequent liquid discharges from the bowels. I then directed for the patient the following, which, taken in doses of a tea-spoonful every two hours, quickly checked the discharges, and an early convalescence was established, viz.:—

℞—Acetas Plumbi, . . ℈j.

Acetas Morph. . . . 2grs.

Water, . . . . . ℥ij.

Mix.

In those cases in which diarrhœa alone supervened, without disturbance of the stomach, I very generally resorted to either the nitrate of silver and opium, or the emulsion of oil of turpentine and laudanum, and with prompt benefit. The formula for the latter has already been given in another part of this report; and the former was usually proportioned as follows, viz.:—

℞—Nitras Argenti, . . . 7grs.
Pulv. Opii, . . . 20grs.

Mix very thoroughly, and divide into twenty pills, one of which may be taken every three, four, or six hours.

I have very rarely met with a case of the enteric variety of typhoid or continued fever, during the first three weeks of its progress, that was not controlled by a judicious and steady use of the foregoing remedies, aided by occasional diuretics, such as the spirits of nitre dulcis and acetate of ammonia; the frequent sponging of the cutaneous surface with cold or tepid water, according to the degree of febrile heat; and the pretty free use of beef-tea or other animal broth, well salted, and sweet milk boiled with wheat-flour, for nourishment.

In the hospital, where we frequently have cases brought in from the poorer classes, in the second and third weeks after the commencement of the fever, with the tongue red at the edges and tip, the middle covered with a dry and brown coat, the whole mouth dry; the mind dull and wandering; the pulse from one hundred to one hundred and twenty; skin dry and harsh; the abdomen tympanitic, and from three to six copious and thin brownish discharges from the bowels every twenty-four hours, I often am obliged to give a tea-spoonful of the emulsion of oil of turpentine and laudanum every three or four hours, with a pill of the nitrate of silver and opium between, and continue them three or four days before the abdominal symptoms are relieved—as indicated by the more moist tongue; the less distended and tympanitic condition of the abdomen; the less frequent and more natural appearance of the discharges; and the improved sensibility of the patient. When these changes had taken place, it was sufficient to continue either one of these remedies, at intervals of four or six hours, until the alvine discharges became entirely natural in consistence and frequency. In several patients, who had become much debilitated, after the tongue and mouth had lost their dryness, it was found useful to add two grains of the tanate of quinine to each dose of the turpentine and laudanum emulsion.

But, as already stated, a large proportion of the cases of continued fever that occurred during the past year, presented, sooner or later, symptoms of pulmonary inflammation or engorgement sufficient to require marked variations in the treatment. When such complications existed in the early stage of the disease, indicated by a mixture of dry bronchial ronchi with a crepitant or sub-crepitant rale, and some dulness on percussion, I generally commenced the treatment with the following, viz.:—

℞—Hive Syrup, . . . ℥j.

Tinct. Opii et Camph. . ℥jss.

Tinct. Verat. Viride, . . ʒj.

Mix,

And give one tea-spoonful every four hours; also, half-way between these doses, a powder composed of, calomel two grains, and Dover's powder five grains. After the continuance of these remedies twenty-four hours, if no evacuations from the bowels had occurred, the powders were suspended, and a table-spoonful of castor-oil administered. More frequently, however, the bowels were evacuated without the oil, and sometimes too freely; and the patients were found, at the end of the twenty-four or thirty-six hours, with less heat and more moisture of the skin; less dry rales in the chest, and a slower, softer pulse. When this was the case, the veratrum was left out of the cough mixture and the other ingredients continued in the same dose, and at the same intervals as before, while between the doses was given the following, viz.:—

℞—Sub-Murias Hydrarg. 2grs.

Pulv. Opii, . . . 1gr.

Pulv. Antimonialis, . 1gr.

Mix one dose.

If, by continuing these remedies from one to two days, the dry rales entirely disappeared, the cough became less frequent, and the expectoration more free; and especially if the skin and tongue became quite moist, with more feebleness of pulse, and feelings of prostration, a more supporting plan of treatment was resorted to, as follows:—

℞—Hive Syrup, . . . ℥j.

Tinct. Bloodroot, . . ℥ss.

Tinct. Opii et Camph. ℥jss.

Mix,

And give one tea-spoonful every four hours, alternated with a powder of, sulph. quinine two grains, pulv. bloodroot half a grain, pulv. Doveri six grains, mixed. If dulness on percussion remained, as was often the case, especially over the lower and posterior portions of the lungs, blisters were now applied and with much benefit. Most of the cases met with would convalesce under the foregoing treatment in from one to two weeks. But some cases were met with, in which the general febrile symptoms subsided in about the usual length of time; the skin became cool, soft, and moist; the tongue clean and moist; the appetite improved, and the bowels regular; but the pulse remained more frequent than normal and feeble; the respiration feeble and incomplete, with occasional paroxysms of coughing; and considerable dulness on percussion over the middle and lower portions of one or both lungs. The patients gained strength very slowly. The difficulty seemed to be, that the texture of those portions of the pulmonary tissue which had been the seat of engorgement, had become so much impaired as to regain its elasticity with great difficulty. In such instances much advantage was derived from giving, instead of the ordinary cough mixture, a solution of muriate of ammonia, according to the following formula:—

℞—Muriate of Ammonia, . ʒij.

Ext. Liquorice, . . ℥ss.

Boiling Water, . . 1 pint.

Of which one table-spoonful was given every three or four hours; and the powder of bloodroot, quinine, and opium, continued three times a-day.

In a number of cases, more particularly those which were brought under treatment in the advanced stage of the disease, well marked enteric symptoms existed coincidently with those of severe pulmonary congestion. In such, recourse was had to the terebinthinate emulsion, or the nitrate of silver and opium, in addition to the foregoing remedies. When, during the progress of the disease, a low, wandering delirium came on with some *subsultus*, it was diminished and more rest procured by giving each evening a pill of pulv. gum camphor and ext. of hyosciamus.

In four or five cases that came under my care in the second and third weeks of the disease, with extensive pulmonary engorgement, a rapid and compressible pulse, a leaden hue of the face, much restlessness, constant subsultus and delirium, great advantage was derived from the internal use of chloroform and nitrous ether. One of the most forbidding cases of this kind that I saw during the past season, was brought into the Mercy Hospital in the third week of the fever. His extremities were cold; pulse one hundred and forty per minute, and feeble; face bloated and of a leaden hue; tongue dry and brown; breathing short and very noisy, from the rattling of mucus in the larger bronchial tubes; the lateral parts of the chest very dull; extreme subsultus tendinum and constant delirium. I ordered for the patient a mixture of, chloroform ʒij, nitrous ether ℥ij, of which a tea-spoonful or fluid drachm was given every hour, diluted with sugar and water; also a liberal quantity of beef-tea, well salted; and sinapisms to his extremities. The next day, finding the patient decidedly improved, the same chloroform mixture was continued at intervals of once in two hours, with a blister on the chest, and a powder of quinine, bloodroot, and Dover's powder, three times a-day. The patient continued to improve so satisfactorily that little else was given him, and he became entirely convalescent in about ten days after his admission into the hospital.

It was my intention to have included in this report some remarks in regard to dysentery; but the disease prevailed only to a very limited extent, and exhibited no peculiarities requiring special notice: consequently it shall be omitted, because the report has already reached a much greater length than was originally assigned to it.

